# Paternal Resistance Exercise Modulates Skeletal Muscle Remodeling Pathways in Fathers and Male Offspring Submitted to a High-Fat Diet

**DOI:** 10.3389/fphys.2021.706128

**Published:** 2021-09-27

**Authors:** Rebecca Salomão, Ivo Vieira de Sousa Neto, Gracielle Vieira Ramos, Ramires Alsamir Tibana, João Quaglioti Durigan, Guilherme Borges Pereira, Octávio Luiz Franco, Carine Royer, Francisco de Assis Rocha Neves, Ana Carolina Andrade de Carvalho, Otávio Toledo Nóbrega, Rodrigo Haddad, Jonato Prestes, Rita de Cássia Marqueti

**Affiliations:** ^1^Laboratory of Molecular Analysis, Faculty of Ceilândia, Universidade de Brasília, Brasília, Brazil; ^2^Graduate Program in Rehabilitation Sciences, Universidade de Brasília, Brasília, Brazil; ^3^Graduate Program of Sciences and Technology of Health, Universidade de Brasília, Brasília, Brazil; ^4^Institute of Health Sciences – Universidade Paulista, São Paulo, Brazil; ^5^Graduate Program in Health Sciences, Faculdade de Medicine, Universidade Federal do Mato Grosso (UFTM), Cuiabá, Brazil; ^6^Interinstitutional Program of Post-Graduation in Physiological Sciences (UFSCar/UNESP), Department of Physiological Sciences, Universidade Federal de São Carlos, São Carlos, Brazil; ^7^Graduate Program in Genomics Science and Biotechnology, Universidade Católica de Brasília, Brasília, Brazil; ^8^S-Inova Biotech, Graduate Program in Biotechnology, Universidade Católica Dom Bosco, Campo Grande, Brazil; ^9^Laboratory of Molecular Pharmacology, Faculty of Health Sciences, Universidade de Brasília, Brasília, Brazil; ^10^Graduate Program of Medical Sciences, Universidade de Brasília, Brasília, Brazil; ^11^Center for Tropical Medicine, Universidade de Brasília, Brasília, Brazil; ^12^Graduate Program of Physical Education, Universidade Católica de Brasilia, Brasília, Brazil

**Keywords:** exercise, intergenerational, gastrocnemius, atrophy/hypertrophy signaling, proinflammatory cytokines, protein turnover, adipogenic

## Abstract

Although some studies have shown that a high-fat diet (HFD) adversely affects muscle extracellular matrix remodeling, the mechanisms involved in muscle trophism, inflammation, and adipogenesis have not been fully investigated. Thus, we investigated the effects of 8 weeks of paternal resistance training (RT) on gene and protein expression/activity of critical factors involved in muscle inflammation and remodeling of fathers and offspring (offspring exposed to standard chow or HFD). Animals were randomly distributed to constitute sedentary fathers (SF; *n* = 7; did not perform RT) or trained fathers (TF *n* = 7; performed RT), with offspring from mating with sedentary females. After birth, 28 male pups were divided into four groups (*n* = 7 per group): offspring from sedentary father submitted either to control diet (SFO-C) or high-fat diet (SFO-HF) and offspring from trained father submitted to control diet (TFO-C) or high-fat diet (TFO-HF). Our results show that an HFD downregulated collagen mRNA levels and upregulated inflammatory and atrophy pathways and adipogenic transcription factor mRNA levels in offspring gastrocnemius muscle. In contrast, paternal RT increased MMP-2 activity and decreased IL-6 levels in offspring exposed to a control diet. Paternal RT upregulated *P70s6k* and *Ppara* mRNA levels and downregulated *Atrogin1* mRNA levels, while decreasing NFκ-B, IL-1β, and IL-8 protein levels in offspring exposed to an HFD. Paternal physical training influences key skeletal muscle remodeling pathways and inflammatory profiles relevant for muscle homeostasis maintenance in offspring submitted to different diets.

## Introduction

Obesity and its comorbidities represent a global health problem that has become a major social and economic burden worldwide. Overweight and obesity are often causally linked to marked alterations in lifestyle, such as increases in high-energy dietary intake, as well as a high prevalence of physical inactivity. In rodent models, a high-fat diet (HFD) promotes accumulation of intramyocellular lipids that induce muscular lipotoxicity, chronic inflammation, oxidative stress, and glucose metabolism abnormalities (Bollheimer et al., 2012; Trajcevski et al., 2013). Among the relevant signaling pathways, NF-κB has emerged as a critical transcription factor that induces the expression of many proinflammatory cytokines (TNF-α, IL-1β, IL-6, IL-8, and IL-12) involved in the skeletal muscle atrophy process (Carlsen et al., 2009). In addition, NF-κB upregulation becomes evident in many catabolic scenarios associated with an increased ubiquitin-proteasome system and autophagy (Demasi and Laurindo, 2012; Sandri, 2013; Abrigo et al., 2016), which in turn might lead to cell damage and muscle disorders.

The inflammatory condition is also closely related to dysfunctional extracellular matrix (ECM) remodeling in skeletal muscle, corroborating musculoskeletal disorders (Tam et al., 2015). Tam et al. (2015) found downregulation of mRNA levels in collagen I, III, and VI in the gastrocnemius muscle of C57BL/6 mice fed an HFD for 5 weeks, indicating imbalance in ECM homeostasis, whose remodeling is regulated by matrix metalloproteinases (MMPs) essential to maintain a favorable environment for structural support, mechanical stability, and transmission of signals from muscle cells. It has been shown that MMP-2 deficiency promotes abnormal changes in collagen (Martinez-Huenchullan et al., 2017), which is crucial to force transmission among fibers and fascicles in the muscle contraction process (de Sousa Neto et al., 2017). The decrease in MMP-2 activity may also impair the release of local growth factors (TGF-β, CTGF, and IGF-1) and proliferation, differentiation, and migration of satellite cells responsible for muscle hypertrophy (Martinez-Huenchullan et al., 2017). Although some studies have already shown that HFD adversely affects muscle ECM remodeling, the mechanisms involved in the muscle trophism, inflammation, and adipogenesis have not yet been fully investigated. Hence, further studies are required to determine the harmful effects caused by an HFD in skeletal muscle.

Resistance exercise training (RT) is a potent lipolysis mediator, preventing intramyocellular lipid expansion in skeletal muscle, linked to the PPARy-mediated mechanism (Ribeiro et al., 2017). The role of RT on skeletal muscle confers beneficial effects on adipogenic transcriptional factors (CCAAT/enhancer-binding proteins), suppressing proinflammatory factors (Guedes et al., 2020) and abnormal ECM remodeling (Guzzoni et al., 2018). Furthermore, RT protects against obesity-induced muscle atrophy, via activation of the anabolic signaling axis *(IGF1*, *AKT, P70sk6*, and *MyoD)* (Glover and Phillips, 2010). Currently, it is widely accepted that a future generation inherits protective exercise-mediated effects through epigenetics inheritance (Vieira de Sousa Neto et al., 2021). The RT performed by fathers represents a potential regulator of intergenerational inheritance and is a cost-effective strategy to improve the health of the first offspring exposed to an HFD (Vieira de Sousa Neto et al., 2021). Thus, the father’s behavior may modulate key players responsible for skeletal muscle remodeling in the offspring.

Previous evidence demonstrated that paternal RT (8 weeks, three times per week) induced protective effects on the left ventricle and tendon proteome of offspring exposed to an HFD through the increase in abundance proteins involved in cellular survival pathways, tissue integrity maintenance, and overall homeostasis (ECM organization, heart contraction, metabolic processes, antioxidant activity, transport, and transcription regulation) (de Sousa Neto et al., 2020a, b). Likewise, Stanford et al. (2018) showed that voluntary paternal running on a wheel (3 weeks with running distances averaging 5.8 ± 0.4 km/day) suppresses the detrimental effects of paternal HFD on offspring, reducing fat mass and glucose uptake in the gastrocnemius, tibialis anterior, extensor digitorum longus, and soleus, which suggests that paternal programming may regulate developmental heritages of offspring diseases. However, the extent to which paternal RT leads to orchestrated molecular changes in offspring skeletal muscle exposed to different diets has not been investigated. The climbing a ladder provides an alternative means of inducing force overload with advantages when improving rat musculoskeletal properties and neuromuscular function (Hornberger and Farrar, 2004), while voluntary wheel running may have limited effects on muscle hypertrophy process (Legerlotz et al., 2008; Kurosaka et al., 2009).

Skeletal muscle adaptation is a complex process involving crosstalk of multiple regulatory mechanisms and intracellular pathways (Potthoff et al., 2007). Identifying essential genes and proteins involved in signaling inflammation, atrophy/hypertrophy molecules, and ECM remodeling could provide meaningful insights into the offspring muscle dysfunctions. A mechanistic framework of controlled paternal RT effects may yield clinically useful information and design interventions to effectively counterbalance harmful responses inherent to HFD. Thus, whether the father’s lifestyle modulates the musculoskeletal system remains a valid question for muscle health and treatment guidance. The purpose of this study was to investigate the effects of 8 weeks of paternal RT on gene and protein expression/activity of crucial factors involved in muscle inflammation and remodeling of fathers and offspring (offspring exposed to standard chow or HFD). We hypothesized that paternal RT could minimize maladaptive responses at the transcriptional level (inflammatory factors, atrophy-associated genes, and ECM markers), proinflammatory cytokines levels, and ECM protein turnover pathways impaired by HFD.

## Materials and Methods

### Animals and Experimental Groups

All procedures were conducted following the Guide for the Care and Use of Laboratory Animals (National Research Council, 1996) in compliance with the ARRIVE guidelines. The research protocol received approval from the Ethics Committee on Animal Experimentation from the Universidade Católica de Brasília, Brasília (protocol No. 010/13). Initially, fourteen (*n* = 14) 4-month-old *Wistar* rats (*Rattus norvegicus albinus*, weighing 359 g ± 32.4) were obtained from the Central Vivarium of the Faculty of Physical Education of the Universidade Católica de Brasilia. The animals were housed in collective cages (maximum of four rats per cage) and received water and standard feed for rodents *ad libitum* during the experimental period. The animals were randomly distributed into two subgroups: sedentary fathers (SF; *n* = 7; did not perform RT) and trained fathers (TF; *n* = 7; performed RT).

### Paternal Resistance Training

The RT protocol was adapted from Hornberger and Farrar (2004), according to the requirements of this study. In addition, the procedures have been described in previous intergenerational studies (de Sousa Neto et al., 2020a, b). The RT protocol was carried out for 8 weeks, with the climbs performed three times a week (Monday, Wednesday, and Friday) in the afternoon (between 2 and 4 p.m.). Initially, the rats were adapted to the resistance exercise protocol, which required the animals to climb a vertical ladder (1.1 × 0.18 m, 2 cm step, 80% climb angle) with the load apparatus fixed to their tail, via a self-adhesive foam strip wrapped around the proximal portion. The size of the steps meant the animals performed 8–12 movements per climb. In the familiarization phase, the rats were placed at the bottom of the stairs and were stimulated to climb with a weight attached to the tail. If necessary, a stimulus through finger tweezers was applied to the animal’s tail to start the movement. At the top of the stairs, the rats reached a house chamber (20 × 20 × 20 cm) where they rested for 2 min. This procedure was repeated until the animals could voluntarily climb the subsequent set without stimulation (de Sousa Neto et al., 2018). The familiarization sessions were performed three times with an interval of 48 h.

Three days after familiarization, each animal was evaluated to determine its maximum load capacity, which consisted of 4–8 ladder climbs with gradually heavier loads. The initial climb consisted of carrying a load corresponding to 75% of the animal’s body weight. After that, weights of 30 g were added progressively until the rat was unable to complete the climb. Failure to climb was determined when the animal was unable to complete the climb after three successive tail stimuli. The highest load carried to the top of the stairs was considered the maximum load capacity of the rat for that specific training session. After defining the maximum load capacity, the training sessions comprised eight climbs (TF), two sets with each load of 50, 75, 90, and 100% of the animal’s maximum load capacity. Average feed intake was measured for each experimental group 24 and 48 h after the first RT session and 72 h after the second RT session.

In each week, respecting the progressive overload principle of the training, 30 g (approximately 13% of the animal’s body weight) were added, referring to the weight of the previous week with 100% of the maximum loading capacity, in order to guarantee the desired stress resulting from the exercise.

### Offspring Groups

The offspring came from mating with sedentary females. The estrous cycle of the females was checked daily after completing the RT. The male rats were mated with primigravidae females during the proestrus phase, housing together for two consecutive days with free access to a control diet (Purina^®^, Descalvado-SP, Brazil) water.

After birth, the experimental groups in this study consisted of 28 male pups. The litters were standardized to 7 pups each to avoid litters of different sizes and were left with their mothers for 21 days of breastfeeding. The litters belonging to the same experimental group were descended from different parents.

After weaning, male pups were divided into 4 groups (*n* = 7 per group): offspring from sedentary father submitted either to control diet (SFO-C) or high-fat diet (SFO-HF) and offspring from trained father submitted to control diet (TFO-C) or high-fat diet (TFO-HF). The animals were housed in polypropylene cages with a temperature of 23 ± 2°C and light:dark cycle of 12:12 h. The pups were weighed, in grams, on a digital scale, weekly for 6 months (Filizola^®^, São Paulo, Brazil).

### Offspring Diet

The offspring submitted to the control diet (SFO-C and TFO-C) were fed with standard feed (66.00% carbohydrates, 24.00% protein, and 10.00% lipids, totaling 3.48 Kcal/g, Labina Presence^®^, Paulínia, São Paulo, Brazil) and water *ad libitum*. Females were also kept on the control diet during gestation and lactation periods. The SFO-HF and TFO-HF groups were submitted to the commercially acquired HF diet (20.27% carbohydrates, 19.89% protein, and 59.38% lipids, totaling 5.20 Kcal/g) (Prag^®^ solutions, Biosciences, Jaú, Brazil) and an overload of 200 mL of soft drink (high-fructose corn syrup, caramel coloring, caffeine, and phosphoric acid) per week (100% carbohydrates—21 g, sodium: 10 mg totaling 0.85 Kcal/g), in addition to having free access to water from the 21st day after birth for 6 months. Detailed compositions of the experimental diets were reported previously (de Sousa Neto et al., 2020b).

**FIGURE 1:**
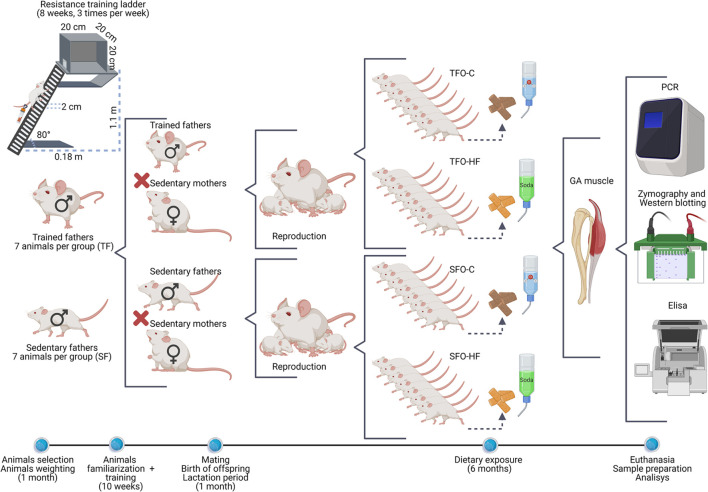
Schematic representation of the experimental design. Illustration of the methodological sequence followed in the intergenerational study.

Some studies have demonstrated the efficacy of this HFD model in body weight gain and adiposity in *Wistar* rats (Goncalves et al., 2018; de Sousa Neto et al., 2020b). Soft drinks for rats are used as a complement and characterization of the HF diet and are an effective strategy to promote the increase in total energy intake and body weight gain in the long term (Messier et al., 2007; Lozano et al., 2016). The offspring were weighed weekly on a digital scale (Filizola, São Paulo, Brazil) until euthanasia. The experimental design of the study is shown in Figure 1.

### Euthanasia

Fathers were euthanized with an intraperitoneal injection of xylazine solution (12 mg/kg body weight) and ketamine (95 mg/kg body weight), 48 h after the training period to avoid the acute effects of exercise. The offspring were euthanized with the same combination of solutions after 6 months of exposure to the HFD. Afterward, the medial gastrocnemius muscles were immediately dissected from each posterior hindlimb and one part was frozen in RNase-free tubes using liquid nitrogen (for qPCR, cytokines, zymography, and western blotting) and then stored at −80°C for further molecular analysis related to skeletal muscle remodeling. An experienced researcher dissected all muscles to prevent contamination of other tissues.

### RNA Extraction

Tissue samples from 28 animals (7 per group) were homogenized in a tube containing five stainless steel balls (diameter, 2.3 mm) (BioSpec Products, Bartlesville, OK, United States), three sharp silicon carbide particles (1 mm) (BioSpec Products), and TRIzol, by shaking using a FastPrep-24 instrument (M.P. Biomedicals, Solon, OH, United States) (Marqueti et al., 2012). In order to thoroughly homogenize the tissue, the shaking process was repeated five times at speed 4. The tubes were kept in ice-cooling for 2 min to avoid RNA degradation between each shaking step. The total RNA was extracted according to the TRIzol method described by Chomczynski and Sacchi (1987). A NanoDrop^®^ spectrophotometer (ND-1000; NanoDrop Technologies Inc., Wilmington, DE, United States) was used to quantify the RNA concentrations in each sample, determining the absorbance rate of 260–280 nm. Subsequently, samples were frozen at −80°C up to purification.

### One Step RT-qPCR

To assess the gastrocnemius muscle gene expression, a total of 100–350 ng of RNA extracted from each sample was converted to cDNA (final volume 20 μL) using GoTaq^®^ Probe 1-Step RT-qPCR (Promega-Cat. A6120X) based on the manufacturer’s protocol. The standard cycling conditions to perform reverse transcription and amplification of samples were: (a) Reverse transcription (1 cycle): 45°C for 15 min; (b) reverse transcriptase inactivation and GoTaq^®^ DNA Polymerase activation (1 cycle): 95°C for 2 min; (c) Denaturation (40 cycles): 95°C for 15 s; and (d) Annealing and extension (40 cycles): 60°C for 1 min.

### Quantitative Real-Time Polymerase Chain Reaction

The quantitative real-time polymerase chain reaction (qRT-PCR) was performed with the aid of the GoTaq^®^ Probe 1-Step RT-qPCR System (Promega—Cat. A6120X). Ten microliters of the GoTaq Probe qPCR Master Mix with dUTP were homogenized with 1 μL of the 20 × primer, an amount of RNA (100–350 ng), 0.4 μL GoScript RT Mix for 1-Step RT-qPCR 1X, and water for a final volume of 20 μL. The qRT-PCR was performed using a QuantStudio 3 Real-Time PCR system (Applied Biosystems) for the following genes (Table 1):

**TABLE 1:** List of tested genes associated with extracellular matrix, inflammation, metabolism, and myogenesis.

mRNA	Code (life technologies)	mRNA	Code (life technologies)
*Actb*	rn00667869	*Murf1*	rn00590197
*Akt1*	rn00583646	*Myod*	rn01511496
*Atrogin1*	rn00591730	*Nfkb*	rn00595794
*Cebpa*	rn00560963	*Ppara*	rn00566193
*Col1a1*	rn01463848	*P70s6k*	rn00583148
*Col3a1*	rn01437681	*Rplp0*	rn03302271
*Ctgf*	rn01537279	*Tgfb1*	rn00572010
*Foxo1*	rn01494868	*Tnfa*	dr03126850
*Gapdh*	rn01775763	*Tweak*	rn01461586
*Igf1a*	rn00710306	*Mmp2*	rn01538170
*Il6*	rn01410330		

*Actb, Actin beta; Akt1, AKT serine/threonine kinase 1; F-box protein 32 (also known as Atrogin1); Cebpa, CCAAT/enhancer binding protein alpha; Col1a1 and Col3a1, respectively, collagen 1 alpha 1 and collagen 3 alpha 1; Ctgf, connective tissue growth factor;Foxo1, forkhead box O1; Igf1, insulin-like growth factor 1; Il6, interleukin 6; Mmp2, matrix metallopeptidase 2; Murf1, tripartite motif containing 63; Myod, myogenic differentiation; Nfkb, nuclear factor kappa beta; Ppara, peroxisome proliferator-activated receptor alpha; ribosomal protein S6 kinase beta-1 (also known as P70s6k), Rplp0, ribosomal protein lateral stalk subunit P0; Tgfb1, transforming growth factor, beta 1; Tnfa, tumor necrosis factor alpha; Tweak, TNF superfamily member 12.*

The normalization of the expression from each target gene occurred through the expression of the constitutive gene RPLP0, which was used as the endogenous control of RNA, due to the lower intra and intergroup variability compared with the other housekeeping genes tested (Beta-actin and Gapdh, also tested in this study). The ΔCt values of the samples were determined by subtracting the average Ct value of the target gene from the average Ct value of the maintenance gene. Next, the ΔΔCt values were calculated by subtracting the ΔCt value from the condition of interest from the ΔCt value from the control condition. Finally, 2^–ΔΔ*Ct*^ values were calculated to present data of relative expression. The sample quantity and the reaction efficiency of each gene analyzed in the present study were determined from a standardization curve, with slope reference parameters equal to −3.3, *R*^2^ = 0.9–1.0, and efficiency above 90%. These genes were chosen because a HFD induces substantial muscle atrophy, inflammation, and dysfunctional ECM remodeling in the skeletal muscle (Tam et al., 2015; Abrigo et al., 2016).

### Cytokine Levels

The quantification of total proteins was performed in a NanoDrop^®^ 2000 spectrophotometer (Thermo Fisher Scientific) using a 260/280 nm relation. Cytokine concentrations were determined by a multiplexed flow cytometry method using bead-based immunoassays developed by the BD Biosciences^®^ (CBA, San Diego, CA, United States), specific for the following mediators: Interleukin (IL)-1beta (IL-1β), IL-6, IL-8, IL-10, IL-12p70, and tumor necrosis factor (TNF-α). Assessments were performed according to the manufacturer’s protocols. Reconstituted cytokine standards and the thawed serum samples were processed using the BD FACS Verse cytometer. Two hundred events or more were acquired for each cytokine bead. Data were analyzed using FCAP software, version 3.0 (BD Biosciences^®^, San Diego, CA, United States). Standard curves for each cytokine were generated using serial dilutions of the mediators supplied, with each sample titrated by linear interpolation. All samples were determined in duplicate to guarantee reliability.

### Gelatin Zymography

The zymography technique was used to measure MMP-2 activity. The gastrocnemius muscle extracts were homogenized and incubated in extraction buffer (10 mM cacodylic acid, pH 5.0; 0,15 M NaCl; 1 μM ZnCl_2_; 20 mM CaCl_2_; 1.5 mM NaN_3_, and 0.01% Triton X-100) with five stainless steel balls (diameter, 2.3 mm) (BioSpec Products, Bartlesville, OK, United States) and three silicon-carbide sharp particles (1 mm) (BioSpec Products) by being shaken in a FastPrep-24 instrument (MP Biomedicals, Solon, OH, United States). Next, the solution was centrifuged for 30 min (13,000 g at 4°C), and the supernatant was reserved. A NanoDrop spectrophotometer (ND-1000; NanoDrop Technologies Inc., Wilmington, DE, United States) was used to quantify protein concentrations. Thirty mg of total protein were loaded into each lane. The samples were concentrated in 30 μg of protein and 10 μL of sample buffer without β-mercaptoethanol (reducing agent) and were resolved by electrophoresis on polyacrylamide gel with SDS and gelatin, in the final concentration of 1 mg/mL. After the run, the gel was washed twice for 20 min in 2.5% Triton X-100 solution to remove the SDS. Next, the gel was incubated in the substrate buffer (Tris- HCl 50 mM pH 8.0, CaCl 2.5 mM; NaN_3_ 0.02%, and ZnCl_2_ 10 mM), at 37°C, for 18 h. Subsequently, the gel was stained with Coomassie Brilliant Blue for 60 min. Afterward, the gel was washed in a solution of methanol 30% and acetic acid 10%. The averages of the band intensities were measured using Image Master 2D Platinum 7.0 software and conducted by a blinded researcher, attenuating possible bias related to this process. Pro and active isoform bands were identified via standard techniques using molecular weight criteria. The bands found in all groups were 72–64 kDa, as proposed by previous studies that evaluated MMP-2 in the skeletal muscle (de Sousa Neto et al., 2017, 2018). The assurance of the analysis accuracy, the gels for zymography were prepared simultaneously using the same solutions. Gels electrophoresis was carried out simultaneously using fresh buffers at the same condition and temperature (inside the fridge) to minimize the variation between the gels. Furthermore, protein normalization, voltage and time during electrophoresis, as well gel staining background, have been carefully standardized.

### Western Blot Assay

Total proteins were extracted from tissues using phosphate buffer saline (PBS 1%, pH 7.4) supplemented with protease inhibitor cocktail (Roche, Germany) with five stainless steel balls (diameter, 2.3 mm) (BioSpec Products, Bartlesville, OK, United States) and three silicon-carbide sharp particles (1 mm) (BioSpec Products) by being shaken in a FastPrep-24 instrument (MP Biomedicals, Solon, OH, United States). After this, the samples were centrifuged (14,000 g for 30 min at 4°C). The supernatant was transferred to a new tube and the protein concentration was determined using a NanoDrop^®^ spectrophotometer (ND-1000; NanoDrop Technologies Inc., Wilmington, DE, United States). The protein (20 μg) was separated using 10% SDS-PAGE and transferred to polyvinylidene difluoride (PVDF) membranes (Merck Millipore). The membranes were blocked using 5% non-fat milk in TBST buffer [200 mM Tris-HCl (pH 7.5), 1.5 M NaCl, 0.1% Tween 20] for 2 h and then incubated with the primary monoclonal antibodies: COL1A1 (Sigma Aldrich, C2456), NF-Kβ p65 (Cell Signaling Technology, #4764), and PPAR-α (Santa Cruz Biotechnology, sc-9000) overnight at 4°C. Next, the membrane was incubated with secondary anti-mouse IgG antibody -HRP (Santa Cruz Biotechnology, Sc -2005) or anti-rabbit IgG antibody -HRP (Cell Signaling Technology, #7074) for 1 h at room temperature. Chemiluminescence was detected using an ECL western blotting detection kit (GE Healthcare, Chicago, IL, United States). Densitometry was performed using ImageJ (National Institutes of Health, Bethesda, MD). The intensity related to the analyzed protein was normalized to the GAPDH band (A1978, Sigma Aldrich). The gels for western blot assay were prepared simultaneously using the same solutions to assure the analysis accuracy. Gels electrophoresis was carried out simultaneously using fresh buffers at the same condition and temperature to minimize the variation between the gels. Furthermore, protein normalization, voltage and time during electrophoresis have been carefully standardized. We also choose the best normalization standard; it was confirmed the primary antibody’s quality (monoclonal), prevented signal saturation and accurately quantifying the signal intensity of the target protein.

### Statistical Analysis

The results are expressed as mean ± standard deviation (SD). The Shapiro-Wilk test was used to determine the normality of data, and the Levene test was used to analyze the homogeneity of the variance. Two-way ANOVA (diet x paternal training) was used to compare the dependent variables between the four groups of offspring. When a significant difference was detected, the Tukey *post hoc* test was applied to identify where the difference occurred. An alpha level of *p* ≤ 0.05 was considered significant. The software GraphPad Prism 8.3 (San Diego, CA, United States) was used for statistical analysis and graphics design. The results from gene expression were presented according (Heinemeier et al., 2007, Marqueti et al., 2018; Barin et al., 2019). The mRNA gene expression was log-transformed for the statistical analyses to obtain normal distribution. Data are presented in graphs as geometric mean ± back-transformed standard error (SE) calculated as follows: Geometric mean = 2^ or 10^ logarithmic mean. The geometric mean at 2^ was used only when the logarithmic value was higher than 1,000. Back-transformed positive SE = 2^ or 10^ (logarithmic mean + logarithmic SE) – geometric mean. Back-transformed negative SE = geometric mean – 10^(logarithmic mean + logarithmic SE). The mRNA data are presented on a logarithmic scale as fold changes relative to the mean of the young sedentary group. For this reason, the values for SF and SFO-C groups are always “1.” Biorender web-based software was used to create the study design and the final figure (License Number QH22U934HD).

## Results

### Effects of High-Fat Diet and Paternal Resistance Training on Offspring Body Weight Gain and Adiposity Index

Offspring exposed to the HFD showed a higher body weight and adiposity index when compared with the offspring exposed to the control diet (*p* = 0.001) following 6 months of diet exposure, based on previously published works (de Sousa Neto et al., 2020a, b). Paternal RT attenuated the body weight gain (TFO-HF = 398.8 ± 8.1 vs. SFO-HF 433.9 ± 34.7; *p* = 0.001) and adiposity index (TFO-HF = 2.63 ± 0.68 vs. SFO-HF = 4.75 ± 0.95) in the offspring submitted to an HFD. No statistical changes in body weight were observed between the TFO-C and SFO-C groups.

### Effects of High-Fat Diet and Paternal Resistance Training on Structural Matrix Proteins, Matrix Remodeling Enzymes, and Growth Factor mRNA Levels in the Gastrocnemius Muscle

**FIGURE 2:**
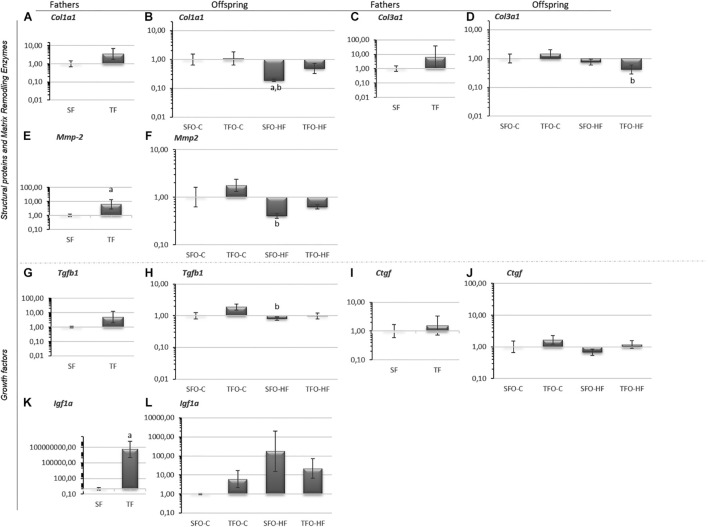
Structural matrix proteins, matrix remodeling enzymes, and growth factor. mRNA levels in the gastrocnemius muscle of fathers and offspring exposed to control diet and HFD. The data are geometric mean ± standard error. The 2^–ΔΔ*CT*^ represents the expression level. **(A,B)** Collagen, type I, alpha 1 (*Col1a1*). **(C,D)** Collagen, type III, alpha 3 (*Col3a1*). **(E,F)** Matrix metalloproteinase-2 (*Mmp2*). **(G,H)** Transforming growth factor-beta 1 (TGF-β1). **(I,J)** Connective tissue growth factor (*Ctgf*). **(K,L)** Pro-insulin like growth factor IA (*Igf1a*). SF, sedentary fathers; TF, trained fathers; SFO-C, offspring from sedentary fathers, exposed to control diet; TFO-C, offspring from trained fathers exposed to control diet; SFO-HF, offspring from sedentary fathers exposed to high-fat diet; TFO-HF, offspring from trained fathers exposed to a high-fat diet. Statistically significant differences compared to: ^*a*^SFO-C; ^*b*^TFO-C; *p* ≤ 0.05 (*n* = 7 per group).

There were no differences between sedentary and trained fathers in mRNA levels of *Col1a1, Col3a1, Tgfβ1, Ctgf* (*p* > 0.05; Figures 2A,C,G,I, respectively), while *Mmp2* and *Igf1a* mRNA levels were significantly upregulated in the trained fathers (*p* = 0.03 and *p* = 0.001, Figures 2E,K, respectively). Regarding offspring, C*ol1a1* mRNA levels were significantly downregulated with HFD (SFO-HF vs. SFO-C; *p* = 0.03; Figure 2B). Furthermore, HFD downregulated C*ol3a1* mRNA levels in the offspring from trained fathers (TFO-HF vs. TFO-C group; *p* = 0.04, Figure 2D). However, paternal RT did not modulate structural matrix proteins, matrix remodeling enzymes, and growth factor mRNA levels in the offspring (*p* > 0.05; Figures 2F,H,J,L, respectively).

### Effects of High-Fat Diet and Paternal Resistance Training on Synthesis and Atrophy Pathway mRNA Levels in the Gastrocnemius Muscle

**FIGURE 3:**
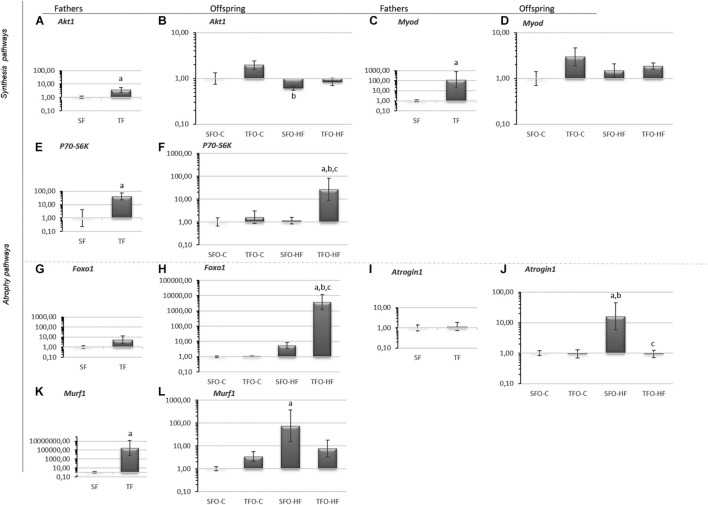
Synthesis and atrophy pathway mRNA levels in the gastrocnemius muscle of fathers and offspring exposed to control diet and HFD. The data are geometric mean ± standard error. The expression level is represented by the 2^–ΔΔ*CT*^. **(A,B)** Protein kinase B (*Akt1*). **(C,D)** Myoblast determination protein 1 (*Myod*). **(E,F)** Ribosomal protein S6 kinase beta-1 (*P70s6k*). **(G,H)** Forkhead Box O1 (*Foxo1*1). **(I,J)** Atrogin 1 (*Atrogin 1*). **(K,L)** Muscle RING-finger protein-1 (*Murf1*). SF, sedentary fathers; TF, trained fathers; SFO-C, offspring from sedentary fathers, exposed to control diet; TFO-C, offspring from trained fathers exposed to control diet; SFO-HF, offspring from sedentary fathers exposed to high-fat diet; TFO-HF, offspring from trained fathers exposed to a high-fat diet. Statistically significant differences compared to: ^*a*^SFO-C; ^*b*^TFO-C; ^*c*^SFO-HF, *p* ≤ 0.05 (*n* = 7 per group).

*Akt1, MyoD, P70S6K*, and *Murf1* mRNA levels were increased after RT in trained fathers (*p* = 0.01; Figures 3A,C,E,K, respectively). Despite the difference presented in the Akt graph (SFO-HF vs. TFO-C; *p* = 0.01; Figure 3B) it did not cause any significant comparison. No changes were observed in fathers for mRNA levels of *Foxo1* (*p* > 0.05; Figure 3G) and *Atrogin1* (*p* > 0.05; Figure 3I). Regarding offspring, *Atrogin1* and *Murf1* mRNA levels were significantly upregulated with HFD (SFO-HF vs. SFO-C; *p* = 0.008 and *p* = 0.01; Figures 3J–L). Paternal RT upregulated *P70s6k* and *Foxo1* mRNA levels in the TFO-HF group when compared to the SFO-HF group (*p* = 0.03 and *p* = 0.001; Figures 3F,H, respectively). Additionally, paternal RT mitigated the HFD-associated increase in *Atrogin1* mRNA levels of offspring (TFO-HF vs. SFO-HF; *p* = 0.007; Figure 3J). No changes were observed in mRNA levels of *MyoD* between offspring groups (*p* > 0.05; Figure 3D).

### Effects of the High-Fat Diet and Paternal Resistance Training on Inflammatory Pathways, Adipogenic Transcription Factor, and Transcription Factor Regulator of Lipid Metabolism mRNA Levels in the Gastrocnemius Muscle

**FIGURE 4:**
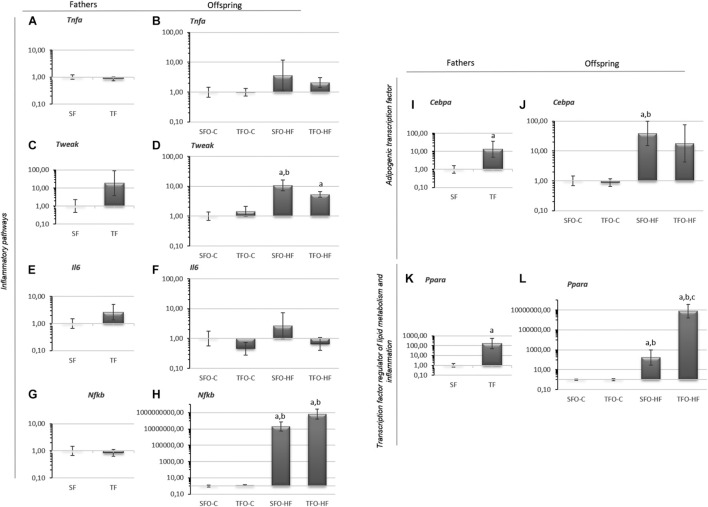
Inflammatory pathways, adipogenic transcription factor and transcription factor regulator of lipid metabolism mRNA levels in the gastrocnemius muscle of fathers and offspring exposed to control diet and HFD. **(A,B)** Tumor necrosis factor alpha (*Tnfa*). **(C,D)** Tumor necrosis factor ligand superfamily member 12 (*Tweak*). **(E,F)** Interleukin-6 (*Il6*). **(G,H)** Factor nuclear kappa B (*Nfkb*). **(I,J)** CCAAT/enhancer binding protein (*Cebpa*). **(K,L)** Peroxisome proliferator-activated receptor (*Ppara*). The data are geometric mean ± standard error. The expression level is represented by the 2^–ΔΔ*CT*^. SF, sedentary fathers; TF, trained fathers; SFO-C, offspring from sedentary fathers, exposed to control diet; TFO-C, offspring from trained fathers exposed to control diet; SFO-HF, offspring from sedentary fathers exposed to high-fat diet; TFO-HF, offspring from trained fathers exposed to a high-fat diet. Statistically significant differences compared to: ^*a*^SFO-C; ^*b*^TFO-C; ^*c*^SFO-HF, *p* ≤ 0.05 (*n* = 7 per group).

No changes were observed in inflammatory pathway mRNA levels in the fathers (*p* > 0.05; Figures 4A,C,E,G). *Cebpa* and *Ppara* mRNA levels were increased in the trained fathers when compared with sedentary fathers (*p* = 0.04 and *p* = 0.01, respectively; Figures 4I,K). Regarding offspring, no changes were observed in *Tnfa* mRNA levels (*p* > 0.05; Figure 4B). *Tweak*, *Cebpa*, and *Nfkb* mRNA levels were significantly upregulated with HFD (SFO-HF vs. SFO-C; *p* = 0.01; Figures 4D,J,H, respectively). Paternal RT upregulated *Ppara* mRNA levels in the TFO-HF group when compared to the SFO-HF group (*p* = 0.0001, Figure 4L). No changes were observed in mRNA levels of *Il6* between offspring groups (*p* > 0.05; Figure 4F).

### Effects of High-Fat Diet and Paternal Resistance Training on Proinflammatory and Anti-Inflammatory Cytokines Levels in the Gastrocnemius Muscle

**FIGURE 5:**
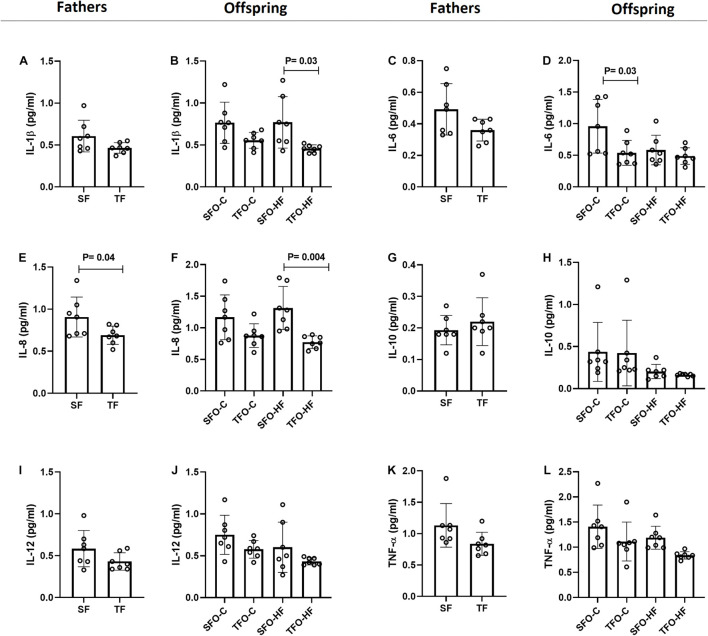
Pro-inflammatory and anti-inflammatory cytokine levels in the gastrocnemius muscle of fathers and offspring exposed to control diet and HFD. The data are mean ± standard deviation. **(A,B)** Interleukin 1β (IL-1β). **(C,D)** Interleukin-6 (IL-6). **(E,F)** Interleukin 8 (IL-8). **(G,H)** Interleukin 10 (IL-10). **(I,J)** Interleukin 12 (IL-12). **(K,L)** Tumor necrosis factor alpha (Tnf-α). SF, sedentary fathers; TF, trained fathers; SFO-C, offspring from sedentary fathers, exposed to control diet; TFO-C, offspring from trained fathers exposed to control diet; SFO-HF, offspring from sedentary fathers exposed to high-fat diet; TFO-HF, offspring from trained fathers exposed to a high-fat diet (*n* = 7 per group).

HFD did not modulate proinflammatory and anti-inflammatory cytokine levels in the offspring’s gastrocnemius muscle (*p* > 0.05; Figure 5). However, paternal RT decreased IL-6 levels in the TFO-C group when compared to the SFO-C group (*p* = 0.03; Figure 5C). Moreover, paternal RT decreased the IL-1β levels in the offspring exposed to HFD. (TFO-HF vs. SFO-HF; *p* = 0.03; Figure 5A). Of note, IL-8 levels changed similarly in fathers and offspring, showing downregulation in the groups under the RT regimen (TF vs. SF; *p* = 0.04 and TFO-HF vs. SFO-HF; *p* = 0.004; Figures 5E,F, respectively). No changes were observed in IL-10, IL-12, and TNF-α levels between offspring groups (*p* > 0.05; Figures 5H,J,L, respectively).

### Effects of High-Fat Diet and Paternal Resistance Training on MMP-2 Activity in the Gastrocnemius Muscle

**FIGURE 6:**
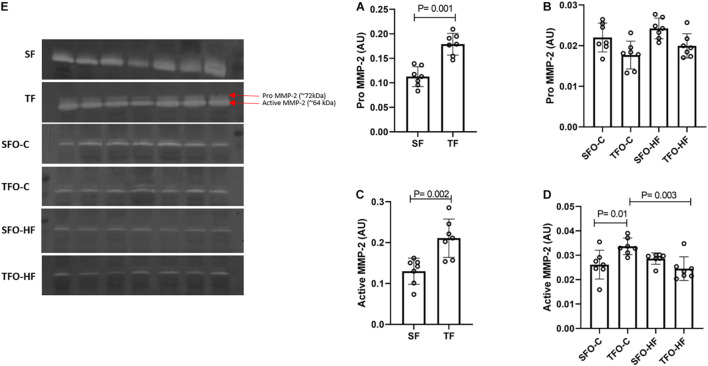
MMP-2 activity in the gastrocnemius muscle of fathers and offspring exposed to control diet and HFD. Optical densitometry of zymographic bands of MMP-2 in arbitrary units (AU). The data are mean ± standard deviation. **(A)** Pro MMP-2 activity in fathers. **(B)** Pro MMP-2 activity in offspring. **(C)** Active MMP-2 activity in fathers. **(D)** Active MMP-2 activity in offspring. **(E)** Optical densitometry of zymographic bands. SF, sedentary fathers; TF, trained fathers; SFO-C, offspring from sedentary fathers, exposed to control diet; TFO-C, offspring from trained fathers exposed to control diet; SFO-HF, offspring from sedentary fathers exposed to high-fat diet; TFO-HF, offspring from trained fathers exposed to a high-fat diet (*n* = 7 per group).

The pro and active MMP-2 activity increased in the gastrocnemius muscle of fathers in response to RT (TF vs. SF; *p* = 0.001 and *p* = 0.002, respectively, Figures 6A,C). No changes were observed in the pro-MMP-2 activity of offspring (*p* > 0.05; Figure 6B). The active MMP-2 activity was significantly decreased with HFD in the offspring from trained fathers (TFO-C vs. TFO-HF; *p* = 0.003; Figure 6D). On the other hand, paternal RT increased active MMP-2 activity in the TFO-C group when compared to the SFO-C group (*p* = 0.01; Figure 6D). MMP-9 activity was not detected by zymography.

### High-Fat Diet and Paternal Resistance Training on Col1, NFκ-B, and PPAR-α Protein Levels in the Gastrocnemius Muscle

**FIGURE 7:**
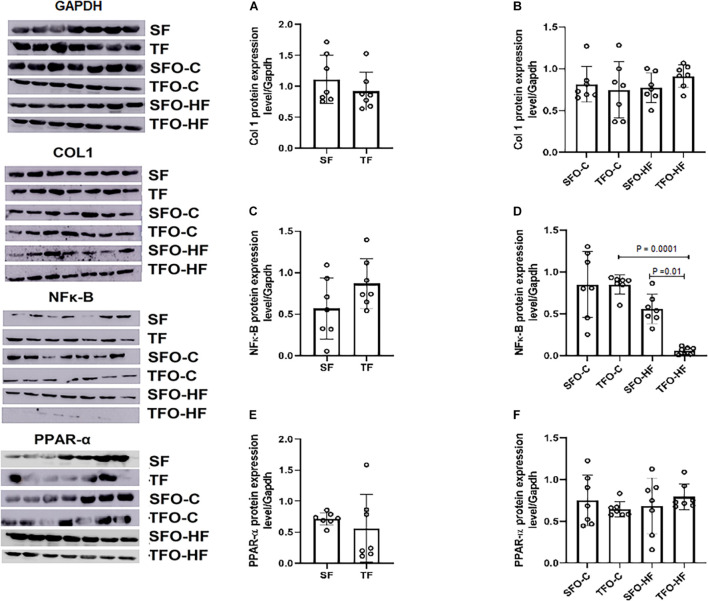
Collagen type I, NF-κB, and PPAR-α protein expression in the gastrocnemius muscle of fathers and offspring exposed to control diet and HFD. The data are mean ± standard deviation. **(A)** Col 1 protein expression level/Gapdh in the fathers. **(B)** Col 1 protein expression level/Gapdh in the offspring. **(C)** NF-κB protein expression level/Gapdh in the fathers. **(D)** NF-κB protein expression level/Gapdh in the offspring. **(E)** PPAR-α protein expression level/Gapdh in the fathers. **(F)** PPAR-α protein expression level/Gapdh in the offspring. SF, sedentary fathers; TF, trained fathers; SFO-C, offspring from sedentary fathers, exposed to control diet; TFO-C, offspring from trained fathers exposed to control diet; SFO-HF, offspring from sedentary fathers exposed to high-fat diet; TFO-HF, offspring from trained fathers exposed to a high-fat diet (*n* = 7 per group).

There were no differences between sedentary and trained fathers in Col1, NFκ-B, and PPAR-α protein levels in the gastrocnemius muscle (*p* > 0.05.; Figures 7A,C,E, respectively). HFD did not modulate Col1, NFκ-B, and PPAR-α protein levels in the offspring from sedentary fathers (SFO-HF vs. SFO-C *p* > 0.05; Figures 7B,D,F, respectively). Intriguingly, the TFO-C group displayed higher levels of NFκ-B when compared to the TFO-HF group (*p* = 0.0001; Figure 7D). However, paternal RT decreased the NFκ-B protein level in offspring exposed to HFD (TFO-HF vs. SFO-HF; *p* = 0.01; Figure 7D). Supplementary Data 1 shows *p*-values of factors (diet and paternal training) and interaction.

## Discussion

The present study was designed to explore the role of pre-conceptional paternal RT on essential molecules involved in the skeletal muscle remodeling of offspring exposed to standard chow or HFD. Our results support the initial hypothesis, showing that HFD downregulated collagen mRNA levels, while upregulating inflammatory and atrophy pathways and adipogenic transcription factor mRNA levels in the offspring gastrocnemius muscle. It was hypothesized that these two findings combined might contribute to the impaired functional integrity of muscle. On the other hand, we demonstrated that paternal RT increased MMP-2 activity in offspring exposed to a control diet, accompanied by a decrease in IL-6 levels. More significantly, paternal RT upregulated *P70s6k* and *Ppara* mRNA levels, as well as downregulating *Atrogin1* mRNA levels and decreasing NFκ-B, IL-1β, and IL-8 protein levels in offspring exposed to HFD. These data support that paternal RT might modulate key skeletal muscle remodeling pathways and inflammatory profiles in offspring submitted to different diets, which may be relevant for cellular function, longevity, and muscle homeostasis maintenance. Our findings raise valuable insights into the molecular mechanisms involved in the intergenerational impacts of paternal exercise on the musculoskeletal system of offspring. This molecular approach progresses the current knowledge on muscle biology.

Effects of resistance training on gene and protein expression/activity involved in muscle inflammation and remodeling of fathers.

Skeletal muscle is a highly plastic organ that plays a pivotal role in physical maintenance and metabolic health (Blaauw et al., 2013). Strategies focused on increasing the quality of this tissue are relevant, and RT is an effective non-pharmacological tool to promote positive impacts on the remodeling process. However, the regulatory mechanisms that mediate muscle adaptations are complex (Blaauw et al., 2013). The present molecular analysis comparing the TF group vs. SF group (fathers) reinforces that RT might modulate multiple routes involved in muscle remodeling. We observed that RT upregulated anabolic markers (*Igf1, Akt, P70sk6*, and *Myod*), *Murf1*, *Cebp*a, *Ppara* mRNA levels, and MMP-2 activity in the gastrocnemius muscle in trained fathers, accompanied by a decrease in IL-8 levels, which may suggest that RT induces molecular adaptation through an integrated signs network. Muscle adaptability is mediated by a complex interplay between a myriad of signaling pathways, joined to several transcription and translation factors (Karalaki et al., 2009; Hoppeler, 2016). The pleiotropic effects of RT and the complexity of responses at a molecular level in our study suggest that there is no singular pathway mediating exercise adaptation. Thus, it is important to emphasize that cellular homeostasis is achieved by a delicate balance between multiple pathways required to carry out complex physiological processes.

Effects of high-fat diet on gene and protein expression/activity involved in muscle inflammation and remodeling of offspring.

The increase in high-energy dietary and unbalanced calorie intake are well-known risk factors that negatively affect skeletal muscle functionality (Lee et al., 2015; Herrenbruck and Bollinger, 2020). The harmful changes are linked to the increase in intramyocellular lipids, associated with lipotoxicity, ECM dysfunctional remodeling, and changes in muscle atrophy−related genes (Abrigo et al., 2016). Indeed, HFD downregulated collagen mRNA levels while upregulating inflammatory (*Tweak* and *Nfkb*) and atrophy pathways (*Atrogin1* and *Murf1*), and Cebpa mRNA levels in the offspring gastrocnemius muscle (SFO-HF vs. SFO-C). This result is in agreement with Kumar et al. (2012), who showed that the *Tweak-Nfkb* axis could be involved in protein breakdown and play an important role in regulating *Murf1* and *Atrogin1* genes. These changes might result in a more inflammatory and catabolic phenotype linked to the adipogenesis integrated process, leading to a panel of adverse remodeling. Nevertheless, we found that gene expression changes were not reflected at the level of proteins at the time-point analyzed, probably due to delayed synthesis between mRNA or the protein during state transition, as well as proteins disconnected from the transcripts (Liu et al., 2016). It is known that protein levels are regulated by transcriptional regulatory elements such as enhancers and post-transcriptional control mechanisms (Sadhale et al., 2007). Furthermore, protein turnover is often under the control of the ubiquitin-proteasome system. One possibility that should not be ruled out is that NF-kB might be associated with ubiquitin-proteasome system overexpression by HFD diet (Abrigo et al., 2016), which may have affected protein patterns and function in the present study.

Other results confirm that HFD has considerable adverse effects on skeletal muscle remodeling due to down-regulation of *Col3a1* mRNA levels and MMP-2 activity, accompanied by up-regulation of *Nfkb* and *Foxo1* mRNA levels in the offspring from trained fathers (TFO-HF vs. TFO-C). The present findings seem to point to progression toward a degenerative phenotype, typical features of muscle wasting and weakness. Abnormal muscle remodeling may impact cell activity, tissue polarity, and muscle repair, inducing chronic disease initiation or progression. Regarding plausible mechanisms, Abrigo et al. (2016) explained that HFD decreases multipotent mesenchymal stromal cells through complex crosstalk of inflammation, oxidative stress, satellite cell dysfunction, and myonuclear apoptosis which, in turn, induces an atrophic effect on skeletal muscle. Furthermore, the adipogenesis process also leads to inflammation and atrophy in muscle cells during obesity (Pellegrinelli et al., 2015), which explains an increase in the *Cebpa*, *Tweak*, and *Nfkb*, *Atrogin1*, and *Murf1* mRNA levels simultaneously in the SFO-HF group.

Effects of paternal resistance training on gene and protein expression/activity involved in muscle inflammation and remodeling of offspring exposed to control diet.

An exciting result was that paternal RT upregulated MMP-2 activity while downregulating *Il6* levels in offspring exposed to the control diet. This suggests that the father’s lifestyle can imply changes in ECM remodeling and the inflammatory profile when offspring are not exposed to a harmful diet. The increase in MMP-2 activity contributes to the remodeling of fibers and connective tissue during normal homeostasis (Chen and Li, 2009). MMP-2 is involved in the morphogenesis and angiogenesis process (de Sousa Neto et al., 2018), playing a critical role in differentiation, muscle repair, and regeneration (de Sousa Neto et al., 2017; Martinez-Huenchullan et al., 2017). Hojman et al. (2019) reported that any ECM alteration initiates the membrane conformational changes needed to release the IL-6-containing intrafibrillar vesicles. Therefore, it seems reasonable to speculate that optimal muscle remodeling modulates inflammation adjustment, considered substantial for muscle adaptation mechanisms. Additional studies should be carried out to test this hypothesis and clarify the causal link between these two biological phenomena. de Sousa Neto et al. (2020b) reported that paternal RT induces modifications at the protein level in the left ventricle of eutrophic offspring. However, this modulation was more prominent in the tendon when the offspring were subjected to HFD (de Sousa Neto et al., 2020a). Therefore, these results support the idea that molecular responses in the offspring might differ between tissue types.

The results of the present study are in agreement with Lira et al., 2009, who demonstrated the potential exercise effect on downregulation of IL-6 levels in healthy rat skeletal muscle. Regarding possible physiological significance, decreased muscle-derived IL-6 production may work to inhibit the effects of pro-inflammatory cytokines, as well as contributing to glucose homeostasis maintenance and lipid metabolism (Lira et al., 2009; Munoz-Canoves et al., 2013). It is therefore possible to speculate that, as well as the attenuation of HPD-associated chronic inflammation, paternal RT may also lead to a decreased risk of the incidence of various diseases associated with an offspring sedentary lifestyle, considering that even in a eutrophic condition (i.e., TFO-C vs. SFO-C) it shows a significant anti-inflammatory effect.

Effects of paternal resistance training on gene and protein expression/activity involved in muscle inflammation and remodeling of offspring exposed to a high-fat diet.

Another novelty of our study was that paternal RT upregulated *P70s6k*, effective in mitigating the HFD-associated increase in *Atrogin1* mRNA levels in offspring exposed to HFD, which can inhibit protein degradation and promote synthesis. Possibly, these adaptations are important to alleviate musculoskeletal damage, the inflammatory microenvironment, and nuclear apoptosis (Abrigo et al., 2016). Thus, paternal RT should be considered an important component to combat skeletal muscle atrophy with HFD.

Remarkably, short (3 weeks) and long-term voluntary wheel protocol (12 weeks) proved to be capable of suppressing the effects of a paternal high-fat diet on offspring, reversing impairment in glucose intolerance, fat mass, and glucose uptake in skeletal muscles (Krout et al., 2018; Stanford et al., 2018). This protection occurred through increased expression of insulin signaling pathways (GLUT4, IRS1, and PI3K) in the skeletal muscle of the offspring (Krout et al., 2018). However, voluntary wheel running possibly has a more considerable aerobic component when compared to weighted stair climbing, which can limit the effects on muscle hypertrophy as previously reported (Legerlotz et al., 2008; Kurosaka et al., 2009). Climbing a ladder provides an alternative means of inducing force overload with advantages when improving musculoskeletal properties and neuromuscular function (Hornberger and Farrar, 2004), and consequent signaling pathway activation that controls skeletal muscle hypertrophy. We consider that the current RT protocol (8 weeks, three times per week) should receive special attention since might open new avenues for preventing and treating muscle wasting in metabolic and neuromuscular diseases in offspring exposed to the risk factors.

Curiously, we found that paternal RT upregulated *Foxo1* mRNA levels in the offspring exposed to HFD. Although *Foxo1* exacerbated activation associated with muscle atrophy, this protein can exhibit ambivalent and pleiotropic functions (Seiler et al., 2013; Sanchez et al., 2014). This suggestion corroborates other studies which showed that *Foxo1* modulates the subcellular localization of the membrane fatty acid translocase that permits fatty acid uptake into muscle cells (Kamei et al., 2003; Bastie et al., 2005). Evidence suggests that *Foxo1* can act as a switch for a shift toward lipids instead of glucose as a fuel substrate under HFD conditions (Constantin-Teodosiu et al., 2012; Sanchez et al., 2014). Additionally, *Ppara* may contribute to adaptive changes in fatty acid metabolism in response to overexpressed *Foxo1* (Constantin-Teodosiu et al., 2012), supporting our TFO-HF group findings. Together, our results suggest that paternal RT can orchestrate factors that might contribute to muscle fat oxidation in the offspring.

**FIGURE 8:**
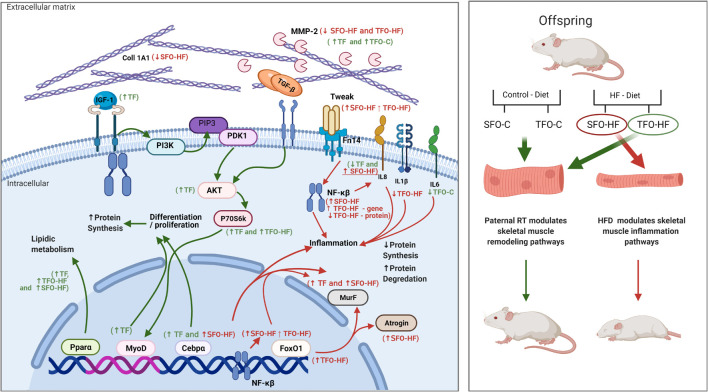
Overview of skeletal muscle remodeling pathways in the gastrocnemius muscle of fathers and male offspring exposed to control diet and HFD.

In this sense, paternal RT also upregulated *Ppara* mRNA levels, besides decreasing NFκ-B and IL-8 protein levels in offspring exposed to HFD, implying a protective factor against the undesired effects of HFD. Upregulated *Ppara* reduces triglyceride levels by increasing lipolysis, contributing to anti-inflammatory activity and metabolic improvements (Gross et al., 2017; Wang et al., 2020). PPAR-α activation interacts with a diverse group of molecules (Viswakarma et al., 2010); for example, PPAR-α inhibits the NF-κB pathway and reduces inflammatory markers, such as IL-1β and IL-8, which in turn suppress atrophy process at multiple levels (Rigamonti et al., 2008). Recently, several plausible studies have revealed that these potent proinflammatory cytokines are involved in calcium homeostasis, mitogenesis, inhibition of angiogenesis, chemotaxis, neutrophil degranulation, and leukocyte activation, which might be an essential contributor to the inflammatory mechanism (Pernhorst et al., 2013; Chuang and Khorram, 2014; Kofanova et al., 2019). Considering our findings, paternal RT may attenuate lipid storage in the skeletal muscle and modulate inflammatory status through IL-1β and IL-8 levels. This result is in agreement with the decrease in *Atrogin1* mRNA levels observed in the TFO-HF group, since it has been demonstrated that IL-1β and IL-8 also play a vital role in atrophy signaling (Callaway et al., 2019). A schematic representation of the intracellular and extracellular environment demonstrates the up and downregulation, and the role of the main genes and proteins identified in this study (Figure 8).

The canonical signaling pathways that induce NF-kB activation converge on the IkB kinase (IKK) complex, which is responsible for IkB phosphorylation and is essential for subsequent translocation of active NF-κB from the cytoplasm to nucleus (Lawrence, 2009). In addition, Toll like receptor (TLR) stimulation and reactive oxygen species (ROS) inherent to HFD provoke activation of multiple signaling pathways, including Ikb/NF-kB axis, JNK/AP-1 signaling, and MAP kinases (ERK1/2, JNK, and p38), promoting subsequent expression of proinflammatory cytokines (Zhang et al., 2016). Alternatively, exercise training decreases TLR expression, while stimulating the expression of antioxidant enzymes and sirtuin 1 (SIRT1), which inhibits NF-κB activity through deacetylation and induces PPARα signaling pathway activation (Gleeson et al., 2006; Liu and Chang, 2018). Collectedly, these adjacent molecular pathways and mutual crosstalk could clarify the possible protective paternal RT effects on skeletal muscle inflammation of offspring in the current study.

Paternal RT did not modulate structural matrix proteins or matrix remodeling enzyme mRNA levels, like Col1 protein level and MMP-2 activity in the gastrocnemius muscle of offspring exposed to HFD, which could be related to similar *Ctgf* and *Tgfb* mRNA levels between the TFO-HF and SFO-HF groups. Skeletal muscle ECM is perturbed under insulin-resistant conditions (Kang et al., 2011, 2013; Graae et al., 2019); however we demonstrated that paternal RT did not decrease the area under the curve (AUC) during the oral glucose tolerance test (de Sousa Neto et al., 2020b), which may have contributed to the limited effects of paternal RT on ECM adaptations in the HFD condition.

It has been shown that paternal exercise in advanced aged sires (>P100) suppresses the effects of paternal high-fat diet on offspring, reversing the observed impairment in glucose tolerance, percentage of fat mass, and glucose uptake in different skeletal muscles of the offspring (Stanford et al., 2018). In the current study, adult rats (4-month-old) affect gene and protein expression in the skeletal muscle of offspring, which suggests that different fathers’ age might modulate the muscle physiology of offspring. Future studies should include the different species of animals and offspring sex and paternal exercise detraining. Furthermore, the effect of lifelong exercise training, not just for a predetermined time intervention or father’s age remains a provocative hypothesis for further investigation. One of the essential aspects of the practical application of this study is to encourage fathers to perform regular exercise throughout life because this might delay the vicious cycle of increased unhealthy risks propagating across generations.

The implementation of paternal RT may become a primary means for combating the ever-growing burden of musculoskeletal system disorders that presently threaten the functional health and wellness of future generations. An epigenetic inheritance mechanistic view could provide crucial clues for public health and clinical practice. Therefore, the tendency or prevalence of muscle abnormalities in the offspring could be partially explained by the father’s environment and lifestyle.

### Limitations

Some limitations of the present study should be highlighted, such as the impossibility of analyzing morphology properties (cross-sectional area, intramyocellular lipids, and ECM markers) and muscle functional assessments (strength and isolated whole-muscle contractility). Our results are also limited to our time diet exposure (i.e., 24 weeks) and male offspring. Further research must include female and male offspring in order to identify specific molecular changes or overlaps between the sexes. The reason for this sex-specificity is uncertain and requires further research. The assessment of only a single point in time is also a considerable limitation. Different time-points from the beginning of dietary intake would be highly valuable in order to clarify the time-course effects of other diets on molecular responses. Non-coding RNA and DNA methylation in the sperm are major routes, which are probably responsible for transmitting the epigenetic landscape from fathers to future generations (Kusuyama et al., 2020; Vieira de Sousa Neto et al., 2021). Additional studies are required to evaluate the link between the sperm epigenetic profile and offspring skeletal muscle remodeling pathways.

## Conclusion

In summary, the father’s lifestyle and the offspring’s diet distinctly modified the key genes and protein expression involved in the muscle inflammation and remodeling pathways. HFD activates inflammatory factors, which in turn might induce dysregulation of signaling pathways associated with the ECM structure and balance between muscle synthesis/breakdown. Interestingly, paternal RT is a critical factor capable of reprogramming different remodeling pathways regardless of the offspring’s diet. In particular, MMP-2 and IL-6 for offspring exposed to the control diet, and the synthesis/atrophy pathways, PPAR-α, and NFκ-B/cytokine axis for offspring submitted to HFD are relevant candidates modulated by paternal RT that may become potential therapeutic targets for treating skeletal muscle disorders. Of relevance, these critical molecules could be contributing toward a muscle-healthy phenotype in the offspring. We believe our findings provide a significant advance to map key paternal exercise-regulated pathways in offspring skeletal muscle.

## Data Availability Statement

The raw data supporting the conclusions of this article will be made available by the authors, without undue reservation.

## Ethics Statement

The research protocol received approval from the Ethics Committee on Animal Experimentation from the Universidade Católica de Brasília, Brasília (protocol no. 010/13).

## Author Contributions

RS, IN, RT, JP, and RM conceived and planned the design of the experiments. RS, IN, GR, RT, and AC performed the experiments, designed the figures, and analyzed the data. RS, IN, GR, JP, and RM interpreted the results and worked on the writing of the manuscript. JD, GP, ON, OF, CR, FN, and RH involved in planning and supervising the work, also contributed to reagents, materials, and analysis tools, implementation of the research and provided critical feedback of the manuscript. All authors discussed the results and contributed to the final manuscript.

## Conflict of Interest

The authors declare that the research was conducted in the absence of any commercial or financial relationships that could be construed as a potential conflict of interest.

## Publisher’s Note

All claims expressed in this article are solely those of the authors and do not necessarily represent those of their affiliated organizations, or those of the publisher, the editors and the reviewers. Any product that may be evaluated in this article, or claim that may be made by its manufacturer, is not guaranteed or endorsed by the publisher.
